# Acquired vulvar lymphangioma circumscriptum after cervical cancer

**DOI:** 10.11604/pamj.2023.44.29.38557

**Published:** 2023-01-16

**Authors:** Jiexia Qiu, Xinrong Chen

**Affiliations:** 1 Zhuji People's Hospital of Zhejiang Province, Shaoxing, China

**Keywords:** Lymphangioma, vulvar, cervical cancer

## Image in medicine

A 72-year-old woman had a history of cervical cancer. She underwent a total hysterectomy twenty years ago and received chemotherapy for one year after surgery. She had lesions with swelling, mild pruritus, and watery secretion on the vulvar surface for two years. Then the lesions gradually increased, causing partial fusion. Histologic examination revealed the vulva had multiple dilated lymphatic vessels in the dermis. The histologic diagnosis was vulvar lymphangioma.

**Figure 1:**
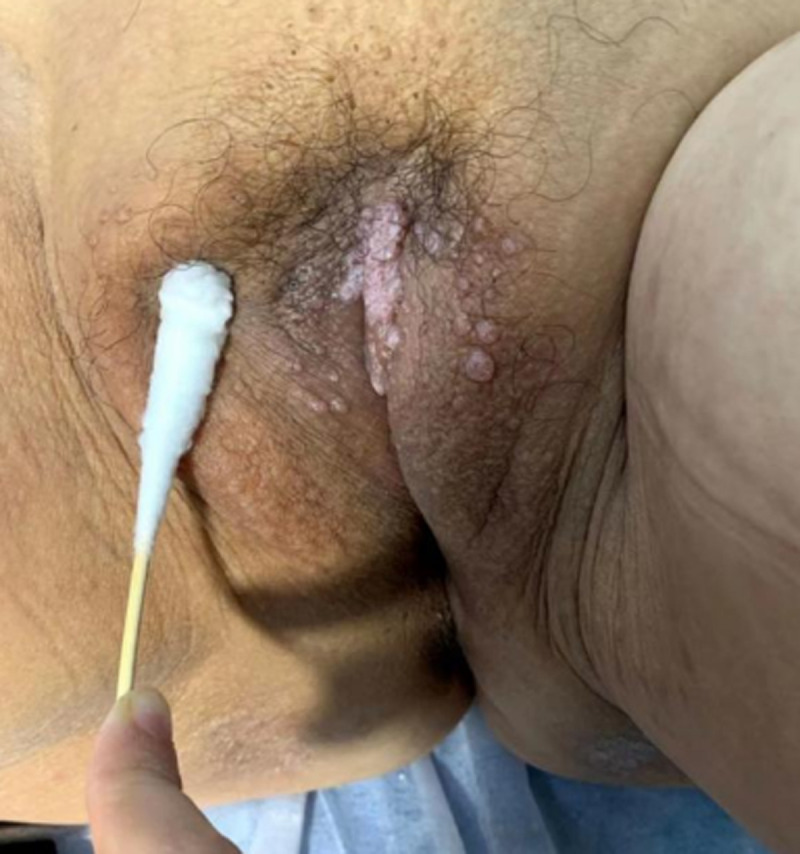
multiple papules and nodules of the vulva resemble false blisters

